# Modifiable determinants of adherence to tyrosine kinase inhibitors in classical myeloproliferative neoplasms: A mixed-methods systematic review protocol

**DOI:** 10.1371/journal.pone.0358485

**Published:** 2026-09-18

**Authors:** Fabiana Coelho Inouye, Vivien Teo, Jessie Clarke, Sofia Georgopoulou, Ana Maria Rosa Freato Gonçalves, Lorena Lôbo de Figueiredo-Pontes, Leonardo Régis Leira Pereira, Sarah Chapman

**Affiliations:** 1 Pharmaceutical Sciences, School of Pharmaceutical Sciences of Ribeirão Preto, University of São Paulo, Ribeirão Preto, Brazil; 2 Institute of Pharmaceutical Science, School of Cancer & Pharmaceutical Sciences, King’s College London, London, United Kingdom; 3 Applied Health Research Group, The Royal Marsden NHS Foundation Trust, London, United Kingdom; 4 Department of Medical Imaging, Hematology, and Clinical Oncology, Ribeirão Preto Medical School, University of São Paulo, Ribeirão Preto, Brazil; Centre de Recherche en Biologie cellulaire de Montpellier, FRANCE

## Abstract

**Introduction:**

Non-receptor Tyrosine kinase inhibitors (TKIs), such as ABL1 and JAK inhibitors, are central to the management of classical myeloproliferative neoplasms (MPNs), yet non-adherence remains frequent and is associated with poorer clinical outcomes. Although adherence-related factors have been explored, the evidence has not been systematically synthesised within a behavioural theory framework to guide the development of effective interventions. This review aims to synthesise evidence on modifiable determinants (barriers and facilitators) influencing adherence to TKI therapy in adults with classical MPNs, and to classify these determinants using the Theoretical Domains Framework (TDF) and Capability, Opportunity, Motivation-Behaviour (COM-B) model.

**Methods:**

This is a mixed-methods systematic review protocol. We will search MEDLINE/PubMed, Embase, CINAHL, LILACS, PsycINFO, and the Cochrane Library from inception to the present, without date restrictions, alongside grey literature sources (OpenGrey, WorldCat, and Google Scholar). Key terms will include myeloproliferative neoplasms, chronic myeloid leukaemia, polycythaemia vera, primary myelofibrosis, tyrosine kinase inhibitors, imatinib, dasatinib, ruxolitinib, and medication adherence. Two reviewers will independently screen studies, extract data, and assess methodological quality using design-appropriate appraisal tools. Quantitative findings will be synthesised narratively and meta-analysed where appropriate. Effect estimates from quantitative studies and qualitative findings (including participant quotations and interpretative data) will be mapped to TDF domains and COM-B components. A mixed-methods synthesis will integrate and triangulate determinants across study designs. Confidence in the evidence will be assessed using GRADE (quantitative) and CERQual (qualitative).

**Conclusion:**

This review will provide a theory-informed synthesis of modifiable determinants of TKI adherence in classical MPNs, supporting the identification of targets for future adherence-enhancing interventions.

**Trial registration:**

**Systematic review registration:** PROSPERO registration number: CRD42024512635.

## Introduction

Myeloproliferative Neoplasms (MPNs) are a heterogeneous group of clonal haematological disorders characterised by the overproduction of myeloid lineage cells [1,2]. Classical MPNs are categorised as either *BCR::ABL1*-positive or *BCR::ABL1-*negative [3]. The *BCR::ABL1* oncogene drives the continuous proliferation of leukaemic cells [4].

Targeted therapies have substantially improved outcomes for patients with MPNs, particularly the use of Tyrosine Kinase Inhibitors (TKIs), a class of orally administered antineoplastic drugs [4]. For the *BCR::ABL1*-positive MPNs, Chronic Myeloid Leukaemia (CML), TKIs of ABL1 including imatinib, dasatinib, nilotinib, ponatinib, and bosutinib are the standard of care. Other TKIs, such Janus kinase (JAK) inhibitors like ruxolitinib and fedratinib, are used to treat *BCR::ABL1*-negative MPNs, including Primary Myelofibrosis (PMF) and Polycythaemia Vera (PV). These drugs have been used for intermediate and high-risk PMF who present inflammatory and/or symptomatic splenomegaly. In PV, they have been used as a second line therapy, particularly in patients who are resistant or intolerant to conventional therapies [3,5]. Although these treatments are not curative, they have significantly improved disease control and, therefore, prolonged survival [6,7].

However, non-adherence to TKI therapy remains a persistent challenge, leading to poorer clinical outcomes, treatment resistance, and therapeutic failure [6,8–11]. According to the World Health Organization, adherence refers to “the degree to which a person’s behaviour corresponds with the agreed recommendations from a healthcare professional” [12].

Non-adherence may be intentional, arising from a conscious decision not to follow treatment recommendations based on personal beliefs or perceptions, or unintentional, resulting from passive or external factors such as forgetfulness, misunderstanding, or lack of access to medication [13,14].

In this context, adherence-related determinants may be classified as modifiable or non-modifiable. Barriers are circumstances that hinder patients from taking medication as prescribed, whereas facilitators are circumstances that make adherence easier. Modifiable determinants include barriers or facilitators that can be influenced by patients, caregivers, or healthcare professionals (within days or weeks), while non-modifiable determinants refer to factors that are not amenable to change within the defined timeframe [15]. Systemic or structural barriers, such as financial toxicity or limited access to healthcare services, will therefore be considered non-modifiable when they require organisational, policy, or system-level changes beyond the defined timeframe.

Reported adherence rates to TKIs vary widely, ranging from 60% to 100%, depending on study design, measurement method, and population [6,8–11]. Factors contributing to non-adherence include the chronic nature of MPNs, self-managed oral therapy, adverse effects, access barriers, and patients’ beliefs about treatment efficacy (5–13). High adherence has been associated with improved event-free survival and cytogenetic response [9].

Although numerous interventions to support medication adherence have been described, their effects have generally been modest and variable across conditions and populations [16,17]. Evidence suggests that even the most effective strategies lead to limited improvements in adherence and clinical outcomes, despite the recognised value of targeted approaches [17]. This variability likely reflects the complex and multifactorial nature of adherence behaviour and the fact that interventions do not always systematically address the most relevant behavioural and contextual barriers [12,14]. In this context, a structured, theory-informed approach is essential to support the systematic identification of key determinants and the design of appropriately targeted adherence interventions [15,18].

However, to date, no previous review has mapped these determinants within established behavioural frameworks, such as the Theoretical Domains Framework (TDF) and the Capability, Opportunity, Motivation-Behaviour (COM-B) model, specifically in relation to TKI adherence among patients with MPNs.

The TDF synthesises constructs from multiple behaviour change theories into 14 domains capturing cognitive, affective, social, and environmental influences on behaviour, while the COM-B model conceptualises behaviour as the result of interactions between Capability, Opportunity, and Motivation, providing a higher-level structure to support synthesis and intervention development [19–21]. Literature also reports the use of these frameworks to identify modifiable determinants of medication adherence across different clinical contexts, supporting their application in evidence synthesis and intervention development [15,18].

Therefore, this mixed-methods systematic review aims to synthesise evidence on modifiable determinants, barriers, and facilitators influencing adherence to TKI therapy among patients with classical MNP. The review will apply the Theoretical Domains Framework (TDF) and the COM-B model to systematically classify adherence-related factors and to inform and facilitate the identification of potential adherence-support strategies in future intervention development.

## Methodology

The protocol for this mixed-methods systematic review is registered in the International Prospective Register of Systematic Reviews (PROSPERO) under number CRD42024512635. The review will be conducted in accordance with the Cochrane Collaboration’s recommendations and follows the guidelines of the Preferred Reporting Items for Systematic Reviews and Meta-Analyses (PRISMA) [22] and the Preferred Reporting Items for Systematic Review and Meta-Analysis Protocols (PRISMA-P) guidelines [23]. The completed PRISMA-P checklist is provided in S1 Check List. In addition, the qualitative synthesis follows the guidelines of the Enhancing Transparency in Reporting the Synthesis of Qualitative Research (ENTREQ) [24].

### Study design

This study is a mixed-methods systematic review. The review will be guided by a research question structured according to PICO (Population/Patient/Problem, Intervention/Indicator, Comparison/Control, and Outcome/Desired Outcome). The inclusion and exclusion criteria are presented in Table 1.

**Table 1:** PICO framework and eligibility criteria.

Category	Inclusion criteria	Exclusion criteria
Year of publication	No date restrictions; full-text available	NA
Study type	Randomised controlled trials (if applicable), prospective and retrospective cohort studies, case-control studies, cross-sectional studies, qualitative studies, and mixed-methods studies	Reviews, protocols, editorials, opinion pieces, conference abstracts without full text, and quantitative intervention studies primarily designed to improve adherence
Language	Portuguese, English, Spanish, or Mandarin	Other languages
Participants	Adults (≥18 years) diagnosed with Chronic Myeloid Leukaemia, Polycythaemia Vera, or Primary Myelofibrosis	Paediatric populations; essential thrombocythaemia; or unrelated diagnoses
Intervention	Treatment with approved TKIs (e.g., imatinib, dasatinib, nilotinib, ruxolitinib, fedratinib, bosutinib, ponatinib, asciminib, radotinib) [26,27]	NA
Comparison	NA	NA
Outcomes	Studies examining modifiable determinants of adherence (barriers or facilitators that can be modified by patients, carers, or healthcare professionals to improve adherence) to TKI therapy. Outcomes may include medication adherence, clinical outcomes, and health-related quality of life	Studies not addressing modifiable determinants of medication adherence.
Adherence measurement	Explicit adherence measure or assessment method (e.g., PDC, self-report scales) required for quantitative studies; not required for qualitative studies	Quantitative studies without clearly defined adherence measurement
Determinants	Studies identifying modifiable behavioural, clinical, social, treatment-related, or system-level determinants of adherence that are amenable to change within days or weeks.	Determinants not clearly identifiable or unrelated to adherence behaviour
Stakeholders	Studies reporting perspectives of patients, caregivers, or healthcare professionals	Studies not involving relevant stakeholder perspectives when examining behavioural determinants
Statistical analysis (quantitative studies)	Inferential statistical analyses examining associations between modifiable determinants of adherence and adherence and/or clinical outcomes.	Studies reporting descriptive data only without analysis of associations

PDC = Proportion of Days Covered; TKIs = Tyrosine kinase inhibitors; NA = Not applicable.

The guiding research question will be: ‘What modifiable factors influence adherence to Tyrosine Kinase Inhibitor therapy in patients with Classical Myeloproliferative Neoplasms (MPNs)?’. Furthermore, the review will investigate how these factors may impact adherence, clinical outcomes, and quality of life, aiming to understand their broader effects.

### Inclusion and exclusion criteria

Studies will be eligible if they include adult patients (≥18 years) with MPNs, diagnosed with Chronic Myeloid Leukaemia, Polycythaemia Vera, or Primary Myelofibrosis, receiving TKIs and reporting modifiable determinants of medication adherence. Studies involving patients with essential thrombocythaemia will be excluded, as TKIs are not currently established as standard therapy for this condition [25]. Studies involving atypical or unclassifiable MPNs will also be excluded unless data for an eligible diagnosis are reported separately.

Quantitative studies will be required to include an explicit adherence measure, while qualitative studies will be required to explore patient, caregiver, or healthcare professional perspectives on adherence-related barriers and facilitators. Full inclusion and exclusion criteria are available in Table 1.

### Search strategy

A comprehensive search will be conducted across the following databases: Cumulative Index to Nursing and Allied Health Literature (CINAHL), Embase®, Latin American and Caribbean Health Sciences Literature (LILACS), Medical Literature Analysis and Retrieval System Online (Medline/PubMed), Cochrane Library, and PsycINFO®. The search strategy will be developed using Boolean operators, descriptors, and synonyms based on the PICO framework, with input from a health sciences librarian (ASO). Controlled vocabulary and free-text terms will be retrieved from PubMed, LILACS, Embase® and CINAHL, selected due to their internationally recognised medical terminologies.

The search strategy will be structured around three main concept groups: (i) diseases, (ii) tyrosine kinase inhibitors, and (iii) medication adherence. Key terms will include, among others, myeloproliferative neoplasms, Chronic Myeloid Leukaemia, Polycythaemia Vera, Primary Myelofibrosis, imatinib, dasatinib, ruxolitinib, tyrosine kinase inhibitors, and medication adherence. The full search strategies for all databases will be provided in (S1 Appendix).

For grey literature searches, simplified free-text terms will be used without Boolean operators, except on platforms that support structured searching (e.g., WorldCat). OpenGrey, WorldCat, and Google Scholar will be searched to identify theses and dissertations. In the case of Google Scholar, results will be screened in order of relevance according to the platform’s default ranking, and the first 100 records retrieved will be assessed for eligibility to ensure feasibility.

The search will include studies involving human participants, published in any language, with no date restrictions. In addition, backward snowballing of reference lists will be conducted to identify further relevant publications, as recommended by Cochrane [28].

### Article selection and data collection process

The lead author (FCI) will retrieve records from all databases included in the search strategy, which was developed with input from a health sciences librarian (ASO). FCI will remove duplicate records prior to screening. The screening process will be conducted in three stages using the Rayyan platform [29] and will involve reviewers FCI, VT, JC, and AF, with SC acting as an independent adjudicator when required.

An initial pilot alignment phase will be coordinated by the lead author (FCI) and conducted with the participation of all reviewers (FCI, VT, JC, and AF) using Rayyan. Prior to screening, FCI will train all reviewers in the use of Rayyan and in the application of the eligibility criteria to ensure consistency. During the pilot phase, a sample of records will be jointly discussed to calibrate screening decisions, and this process will be repeated as needed until satisfactory inter-rater agreement is achieved (κ > 0.7) [30].

Following alignment, title and abstract screening will be conducted in duplicate, with each record screened independently by FCI and one additional reviewer. Disagreements will be resolved through discussion; if consensus is not reached, a third reviewer (JC, VT, or AF) will be consulted, and if disagreement persists, a senior independent reviewer (SC) will provide final adjudication. Full-text screening will follow the same procedure.

To ensure feasibility and accuracy, all stages of study selection were conducted independently in duplicate. Records were allocated according to language expertise when required: FCI reviewed records in Portuguese, Spanish, and English; VT in Mandarin and English; AF in Portuguese, Spanish, and English; and JC in English.

### Data extraction and tabulation

Data extraction, deductive coding, inductive synthesis, and tabulation will be conducted using a structured, multi-stage approach to ensure consistency, transparency, and replicability. For coding purposes, adherence-related data will include statements reported by patients, caregivers, and healthcare professionals, as well as adherence-related themes identified by the original study authors. Procedures will include validation of the extraction form, independent duplicate data extraction, deductive coding using the TDF with subsequent mapping to COM-B, inductive thematic synthesis, and final study mapping. A detailed description of each stage is provided in Table 2.

**Table 2:** Description of data extraction and tabulation procedures.

Stage	Description
**I. Development and validation of the extraction framework**	A standardised data extraction form will be developed by the lead author (FCI) to capture study characteristics, modifiable adherence-related determinants, participant-reported statements (patients, caregivers, and healthcare professionals), author-defined themes, and quantitative outcome measures. The extraction framework will be pilot tested on a subset of studies by the review team to ensure clarity, completeness, and conceptual consistency. Refinements will be implemented through team discussion prior to full extraction.
**II. Independent duplicate data extraction**	All included studies will undergo independent duplicate data extraction. FCI will act as the primary reviewer, with each study independently reviewed by a second reviewer (VT, JC, or AF). Discrepancies will be resolved through discussion, and, where necessary, adjudicated by a third independent reviewer. For mixed-methods studies, qualitative and quantitative components will be extracted separately to allow independent analytical processing within their respective pathways.
**III. Deductive theoretical classification (TDF and COM-B mapping)**	All modifiable adherence-related determinants, irrespective of study design, will be deductively classified within the 14 domains of the TDF. Determinants will include verbatim participant statements, synthesised qualitative findings, author-defined themes, and quantitatively examined variables. A pilot coding phase (approximately 10% of included studies) will be undertaken to develop and calibrate a preliminary codebook (S2 Appendix). Independent coding will be performed, and inter-rater agreement will be monitored (Cohen’s κ ≥ 0.7). During full data extraction and coding, minor refinements to the codebook may be introduced if previously unanticipated classification issues arise. Any modifications will be discussed and agreed upon by the review team to ensure consistency and transparency. Determinants will subsequently be mapped onto COM-B components to support theoretical coherence and cross-study comparability.
**IVa. Inductive thematic synthesis (qualitative analytical pathway)**	Coded modifiable determinants from qualitative and mixed-methods studies will undergo inductive thematic synthesis within each TDF domain, generating higher-order themes and subthemes. Each determinant will then be linked to the corresponding COM-B component to support higher-level conceptual integration. The relative importance of each TDF domain will be assessed using predefined criteria adapted from previous theory-informed evidence syntheses: (a) frequency; (b) level of elaboration; and (c) expressed importance, indicated by emphasis in study discussions, salient participant quotations, or reported quantitative associations [18]. COM-B components will be used to summarise broader behavioural patterns. Disagreements will be resolved through discussion or adjudication.
**IVb. Quantitative synthesis (quantitative analytical pathway)**	Quantitative data examining associations between adherence and modifiable determinants, clinical outcomes, or quality of life will be narratively synthesised and, where at least two studies report comparable outcome measures, meta-analysed using Review Manager (RevMan). Objective and self-reported adherence measures will not be pooled together in the primary analysis (see Data Synthesis). Effect sizes (e.g., odds ratios, risk ratios, or regression coefficients) will be extracted and harmonised where feasible. Pooled estimates will be calculated using random-effects models to account for anticipated methodological and clinical heterogeneity. Statistical heterogeneity will be assessed using the I² statistic, and publication bias will be explored using funnel plots when appropriate. Quantitative determinants will be mapped to their corresponding TDF domains and linked to COM-B components to maintain theoretical integration.
**V. Reporting and cross-manuscript integration**	To ensure methodological depth and analytical clarity, qualitative and quantitative findings will be synthesised separately and may be reported in separate manuscripts, depending on the nature and volume of the included evidence. Both syntheses will retain a shared theoretical framework (TDF and COM-B), enabling conceptual comparability and interpretative integration across publications.

TDF: Theoretical domains framework; COM-B: Capability, Opportunity, Motivation-Behaviour

### Status and timeline

The initial search strategy was conducted in 2024 to assess the feasibility of the review and to support the development of a preliminary coding framework. During this phase, a subset of studies was screened and used to pilot data extraction and familiarisation with the TDF and COM-B, resulting in the development of a preliminary codebook (S2 Appendix).

The search strategy was updated and rerun in 2026. Title and abstract screening, full-text assessment, and data extraction have been completed. Preliminary categorisation of the identified determinants began in June 2026, and coding using the TDF and COM-B frameworks is expected to be completed by August 2026.

Methodological quality assessment and evidence synthesis are planned for August and September 2026. Where feasible, quantitative synthesis, including meta-analysis, will be conducted between September and October 2026. Confidence in the quantitative and qualitative evidence will subsequently be assessed using GRADE and CERQual, respectively. The final results of the review are expected to be available by the end of 2026.

### Dealing with missing data

If essential data or methodological details are not reported, the corresponding authors of the included studies will be contacted to request the missing information or to clarify uncertainties in the study methods. If the required data remain unavailable, the eligibility of the study will be reassessed based on the information provided. Studies with missing data that are still considered eligible will be retained, and the potential impact of the missing information will be addressed in the Discussion section.

### Methodological quality assessment

Methodological quality will be assessed independently and in duplicate. Discrepancies will be resolved through discussion or adjudication by a third reviewer when necessary. Studies will not be excluded based solely on methodological appraisal results; however, identified limitations will be considered during synthesis and certainty assessment.

Design-specific critical appraisal tools will be applied as follows:

- Randomised controlled trials (if identified) will be assessed using the Cochrane Risk of Bias 2 (ROB-2) tool [31], following the Cochrane Handbook [28].
- Observational cohort and case-control studies will be evaluated using the Joanna Briggs Institute (JBI) critical appraisal checklist for cohort studies [32].
- Cross-sectional studies will be assessed using the JBI critical appraisal checklist for analytical cross-sectional studies [33].
- Qualitative studies will be appraised using the Critical Appraisal Skills Programme (CASP) qualitative checklist [34], following the Cochrane Handbook [28].
- Mixed-methods studies will be evaluated using the Mixed Methods Appraisal Tool (MMAT), 2018 version [35], which allows appraisal of qualitative, quantitative, and integration components within a single framework.

Each study will be assessed according to the criteria of the respective tool. Selective reporting within individual studies will be considered as part of the design-specific risk-of-bias assessment. No overall numerical score will be calculated; instead, methodological strengths and limitations will be interpreted narratively. To ensure consistency, a pilot calibration exercise will be conducted on approximately 10% of included studies prior to full appraisal. Inter-rater agreement will be assessed using Cohen’s kappa coefficient (κ ≥ 0.7 will be considered satisfactory).

### Data synthesis

This mixed-methods systematic review will employ a structured, theory-driven analytical framework to synthesise qualitative and quantitative evidence on modifiable determinants influencing adherence to TKI therapy among patients with classical MPNs.

Following independent duplicate data extraction, all modifiable adherence-related determinants, irrespective of study design, will undergo deductive theoretical classification within the 14 domains of the TDF and subsequent mapping onto the COM-B components. Determinants will include participant-reported statements (patients, caregivers, and healthcare professionals), themes identified by original study authors, and quantitatively examined variables related to modifiable adherence determinants. After theoretical classification, analytical pathways will diverge according to study design.

**Qualitative studies.** In the qualitative analytical pathway, coded modifiable determinants derived from qualitative and mixed-methods studies will undergo inductive thematic synthesis within each TDF domain, generating higher-order themes and subthemes reflecting recurring behavioural patterns. The relative importance of domains will be determined using predefined criteria: (a) frequency of contributing studies and barriers/facilitators; (b) level of elaboration, defined by the number and analytical depth of themes/subthemes within each domain; and (c) expressed importance, indicated by emphasis in study discussions, salient participant quotations, or reported quantitative associations [18]. Findings will then be linked to COM-B components to support higher-level conceptual integration.

**Quantitative studies.** In the quantitative analytical pathway, studies examining associations between adherence and modifiable determinants, clinical outcomes, or quality of life will be narratively synthesised and, where at least two studies report comparable outcome measures, meta-analysed using Review Manager (RevMan) software [28]. Effect sizes (e.g., odds ratios, risk ratios, or regression coefficients) will be extracted and harmonised where appropriate and aligned to a consistent direction of effect.

Studies will be eligible for pooling only when they report a compatible effect measure for the same determinant and the same adherence outcome construct, ascertained by methods judged a priori to be comparable. For this purpose, adherence measures will be grouped into three categories: (a) objective pharmacy-based or administrative measures (e.g., medication possession ratio [MPR], proportion of days covered [PDC]); (b) self-report instruments (e.g., Morisky Medication Adherence Scale [MMAS], structured questionnaires); and (c) electronic monitoring (e.g., MEMS). Estimates derived from objective and self-reported measures will not be combined in the primary analysis. Where a determinant is informed only by studies spanning more than one category, a cross-category estimate may be reported as a secondary, exploratory analysis, with measurement modality examined as a subgroup.

Meta-analysis will be undertaken when at least two studies meet these criteria for a given determinant. Determinants informed by a single eligible study, or by studies not meeting these criteria, will be synthesised narratively. Pooled estimates will be calculated using random-effects models to account for expected heterogeneity. Statistical heterogeneity will be assessed using the I^2^ statistic, and publication bias will be explored through funnel plot inspection when feasible. Quantitative modifiable determinants will be mapped to their corresponding TDF domains and subsequently linked to COM-B components to maintain theoretical coherence.

To allow comprehensive exploration of each evidence type while maintaining theoretical coherence, qualitative and quantitative syntheses will be conducted separately and may be reported in separate manuscripts, depending on the nature and volume of the included evidence. Both syntheses will retain the shared theoretical framework (TDF and COM-B), ensuring conceptual comparability and interpretative integration across outputs.

### Assessment of Confidence in the Evidence

The confidence in the body of evidence will be assessed separately for the quantitative and qualitative components, following established frameworks. This approach is adapted from previously published mixed-methods systematic reviews [28].

### Quantitative evidence

The Grading of Recommendations, Assessment, Development and Evaluation (GRADE) [36] approach will be applied to assess the certainty of evidence for associations between specific modifiable determinants (e.g., dosing frequency, formulation characteristics) and adherence outcomes, as well as associations between adherence and clinical outcomes.

Each determinant–outcome relationship will be evaluated independently. Where meta-analysis is conducted, pooled estimates will inform the GRADE assessment. When statistical pooling is not feasible, certainty will be assessed for structured quantitative syntheses where appropriate.

Given that most included studies are expected to be observational, certainty will initially be rated as low and may be downgraded or upgraded based on risk of bias, inconsistency, indirectness, imprecision, and publication bias. Summary of Findings tables will be generated using GRADEpro GDT software.

### Qualitative evidence

Confidence in qualitative findings will be assessed using the Confidence in the Evidence from Reviews of Qualitative Research (CERQual) approach, following the guidance of the Cochrane Qualitative and Implementation Methods Group [37].

Each synthesised theme will be evaluated across methodological limitations, coherence, relevance, and adequacy of data. Confidence will be categorised as high, moderate, low, or very low, with justification provided for downgrading decisions.

### Interpretation

GRADE and CERQual assessments will be interpreted alongside each other within the shared theoretical framework (TDF and COM-B), without generating a combined certainty rating.

### Dissemination

The findings of this review will be disseminated through publication in a peer-reviewed journal and presented at national and international conferences. The results will also be shared through academic networks and social media platforms to reach a broader audience and maximise the impact of the research. Upon completion of the review, the data underlying the findings, including study characteristics, adherence measures, identified determinants, effect estimates, risk-of-bias assessments, and data used in the qualitative and quantitative syntheses, will be made available within the published review and its supplementary materials.

## Discussion

Identifying modifiable factors that influence adherence to TKI therapy will provide a holistic understanding of how behavioural, psychosocial, and clinical determinants interact. Integrating quantitative and qualitative evidence will help inform targeted, theory-driven strategies to improve adherence and, ultimately, enhance the quality of life of individuals with classical MPNs.

## Supporting information

S1 Appendix Database search strategy. (DOCX)

S2 Appendix Codebook. (DOCX)

S1 Check List Prima- P 2015. (DOCX)

## References

[1] Shah NP, Bhatia R, Altman JK, Amaya M, Begna KH, Berman E, et al. Chronic Myeloid Leukemia, Version 2.2024, NCCN Clinical Practice Guidelines in Oncology. Journal of the National Comprehensive Cancer Network. 2024;22:43–69. doi: 10.6004/jnccn.2024.0007 38394770

[2] Nurgat Z, Lawrence M. Management of Myeloproliferative Neoplasms (MPNs). J Oncol Pharm Pract. 2022;28(6):1400–10. doi: 10.1177/10781552221082293 35296179

[3] Arber DA, Orazi A, Hasserjian RP, Borowitz MJ, Calvo KR, Kvasnicka H-M, et al. International Consensus Classification of Myeloid Neoplasms and Acute Leukemias: integrating morphologic, clinical, and genomic data. Blood. 2022;140(11):1200–28. doi: 10.1182/blood.2022015850 35767897 PMC9479031

[4] Suttorp M, Millot F, Sembill S, Deutsch H, Metzler M. Definition, Epidemiology, Pathophysiology, and Essential Criteria for Diagnosis of Pediatric Chronic Myeloid Leukemia. Cancers. 2021;13:798. doi: 10.3390/cancers13040798 PMC7917817 33672937

[5] Weltgesundheitsorganisation. WHO classification of tumours of haematopoietic and lymphoid tissues. Revised 4th edition. Swerdlow SH, Campo E, Harris NL, Jaffe ES, Pileri SA, Stein H, et al., editors. Lyon: International Agency for Research on Cancer; 2017.

[6] Gerds AT, Gotlib J, Ali H, Bose P, Dunbar A, Elshoury A, et al. Myeloproliferative Neoplasms, Version 3.2022, NCCN Clinical Practice Guidelines in Oncology. J National Comprehensive Cancer Network. 2022;20:1033–62. doi: 10.6004/jnccn.2022.0046 36075392

[7] Hochhaus A, Baccarani M, Silver RT, Schiffer C, Apperley JF, Cervantes F, et al. European LeukemiaNet 2020 recommendations for treating chronic myeloid leukemia. Leukemia. 2020;34(4):966–84. doi: 10.1038/s41375-020-0776-2 32127639 PMC7214240

[8] Guglielmelli P, Palandri F, Selleri C, Cilloni D, Mendicino F, Mazza P, et al. Adherence to ruxolitinib, an oral JAK1/2 inhibitor, in patients with myelofibrosis: interim analysis from an Italian, prospective cohort study (ROMEI). Leuk Lymphoma. 2022;63(1):189–98. doi: 10.1080/10428194.2021.1969388 34521299

[9] Jabbour E, Kantarjian H. Chronic myeloid leukemia: 2020 update on diagnosis, therapy and monitoring. Am J Hematol. 2020;95(6):691–709. doi: 10.1002/ajh.25792 32239758

[10] Wei G, Rafiyath S, Liu D. First-line treatment for chronic myeloid leukemia: dasatinib, nilotinib, or imatinib. J Hematol Oncol. 2010;3:47. doi: 10.1186/1756-8722-3-47 21108851 PMC3000369

[11] Marin D, Bazeos A, Mahon F-X, Eliasson L, Milojkovic D, Bua M, et al. Adherence is the critical factor for achieving molecular responses in patients with chronic myeloid leukemia who achieve complete cytogenetic responses on imatinib. J Clin Oncol. 2010;28(14):2381–8. doi: 10.1200/JCO.2009.26.3087 20385986 PMC6366340

[12] Sabaté E. Adherence to long-term therapies: evidence for action. Geneva: World health organization; 2003.

[13] Jimmy B, Jose J. Patient medication adherence: measures in daily practice. Oman Med J. 2011;26:155–9. doi: 10.5001/omj.2011.38 PMC3191684 22043406

[14] National Collaborating Centre for Primary Care (UK). Medicines Adherence: Involving Patients in Decisions About Prescribed Medicines and Supporting Adherence [Internet]. London: Royal College of General Practitioners (UK); 2009 [cited 2026 Feb 23]. http://www.ncbi.nlm.nih.gov/books/NBK55440/. Accessed 23 Feb 2026. 21834197

[15] Prajapati AR, Dima A, Mosa G, Scott S, Song F, Wilson J, et al. Mapping modifiable determinants of medication adherence in bipolar disorder (BD) to the theoretical domains framework (TDF): a systematic review. Psychol Med. 2021;51(7):1082–98. doi: 10.1017/S0033291721001446 34006337 PMC8188530

[16] Haynes RB, McDonald H, Garg AX, Montague P. Interventions for helping patients to follow prescriptions for medications. Cochrane Database Syst Rev. 2002;(2):CD000011. doi: 10.1002/14651858.CD000011 12076376

[17] Schnorrerova P, Matalova P, Wawruch M. Medication adherence and intervention strategies: why should we care. Bratisl Med J. 2025;126:1196–206. doi: 10.1007/s44411-025-00227-0

[18] Bott D, Subramanian A, Edgar D, Lawrenson JG, Campbell P. Barriers and enablers to medication adherence in glaucoma: A systematic review of modifiable factors using the Theoretical Domains Framework. Ophthalmic Physiol Opt. 2024;44(1):96–114. doi: 10.1111/opo.13245 37985237

[19] Atkins L, Francis J, Islam R, O’Connor D, Patey A, Ivers N, et al. A guide to using the Theoretical Domains Framework of behaviour change to investigate implementation problems. Implement Sci. 2017;12(1):77. doi: 10.1186/s13012-017-0605-9 28637486 PMC5480145

[20] Cane J, O’Connor D, Michie S. Validation of the theoretical domains framework for use in behaviour change and implementation research. Implement Sci. 2012;7:37. doi: 10.1186/1748-5908-7-37 22530986 PMC3483008

[21] Michie S, Johnston M, Francis J, Hardeman W, Eccles M. From Theory to Intervention: Mapping Theoretically Derived Behavioural Determinants to Behaviour Change Techniques. Applied Psychology. 2008;57(4):660–80. doi: 10.1111/j.1464-0597.2008.00341.x

[22] Page MJ, McKenzie JE, Bossuyt PM, Boutron I, Hoffmann TC, Mulrow CD, et al. A declaração PRISMA 2020: diretriz atualizada para relatar revisões sistemáticas. Revista Panamericana de Salud Pública. 2022;46:1. doi: 10.26633/RPSP.2022.112 PMC9798848 36601438

[23] Moher D, Shamseer L, Clarke M, Ghersi D, Liberati A, Petticrew M, et al. Preferred reporting items for systematic review and meta-analysis protocols (PRISMA-P) 2015 statement. Syst Rev. 2015;4(1):1. doi: 10.1186/2046-4053-4-1 25554246 PMC4320440

[24] Tong A, Flemming K, McInnes E, Oliver S, Craig J. Enhancing transparency in reporting the synthesis of qualitative research: ENTREQ. BMC Med Res Methodol. 2012;12:181. doi: 10.1186/1471-2288-12-181 23185978 PMC3552766

[25] Grunwald MR, Ritchie EK, Rumi E, Assad A, Hamer-Maansson JE, Yu J, et al. Treatment comparison of hydroxyurea versus ruxolitinib in essential thrombocythaemia: A matched-cohort analysis. EJHaem. 2024;5(4):778–83. doi: 10.1002/jha2.954 39157625 PMC11327719

[26] Thomson RJ, Moshirfar M, Ronquillo Y. Tyrosine Kinase Inhibitors. StatPearls [Internet]. Treasure Island (FL): StatPearls Publishing; 2025 [cited 2026 Feb 23]. http://www.ncbi.nlm.nih.gov/books/NBK563322/. Accessed 23 Feb 2026.

[27] Cohen MH, Williams G, Johnson JR, Duan J, Gobburu J, Rahman A, et al. Approval summary for imatinib mesylate capsules in the treatment of chronic myelogenous leukemia. Clin Cancer Res. 2002;8(5):935–42. 12006504

[28] Higgins JPT, Thomas J, Chandler J, Cumpston M, Page MJ, et al. Cochrane Handbook for Systematic Reviews of Interventions version 6.5 [Internet]. [cited 2026 Feb 23]. www.cochrane.org/handbook. Accessed 23 Feb 2026.

[29] Ouzzani M, Hammady H, Fedorowicz Z, Elmagarmid A. Rayyan-a web and mobile app for systematic reviews. Syst Rev. 2016;5(1):210. doi: 10.1186/s13643-016-0384-4 27919275 PMC5139140

[30] Landis JR, Koch GG. An application of hierarchical kappa-type statistics in the assessment of majority agreement among multiple observers. Biometrics. 1977;33(2):363–74. doi: 10.2307/2529786 884196

[31] Sterne JAC, Savović J, Page MJ, Elbers RG, Blencowe NS, Boutron I, et al. RoB 2: a revised tool for assessing risk of bias in randomised trials. BMJ. 2019;:l4898. doi: 10.1136/bmj.l4898 31462531

[32] Barker TH, Hasanoff S, Aromataris E, Stone JC, Leonardi-Bee J, Sears K, et al. The revised JBI critical appraisal tool for the assessment of risk of bias for cohort studies. JBI Evid Synth. 2025;23(3):441–53. doi: 10.11124/JBIES-24-00103 39177422

[33] Ma L-L, Wang Y-Y, Yang Z-H, Huang D, Weng H, Zeng X-T. Methodological quality (risk of bias) assessment tools for primary and secondary medical studies: what are they and which is better? Military Med Res. 2020;7:7. doi: 10.1186/s40779-020-00238-8 PMC7049186 32111253

[34] Critical Appraisal Skills Programme. CASP Checklist: CASP Qualitative Studies Checklist [Internet]. 2023.[cited 2026 Feb 23]. https://casp-uk.net/casp-tools-checklists/qualitative-studies-checklist/. Accessed 23 Feb 2026.

[35] Hong QN, Pluye P, Fàbregues S, Bartlett G, Boardman F, Cargo M, et al. Improving the content validity of the mixed methods appraisal tool: a modified e-Delphi study. Journal of Clinical Epidemiology. 2019;111:49–59.e1. doi: 10.1016/j.jclinepi.2019.03.008 30905698

[36] Atkins D, Best D, Briss PA, Eccles M, Falck-Ytter Y, Flottorp S, et al. Grading quality of evidence and strength of recommendations. BMJ. 2004;328(7454):1490. doi: 10.1136/bmj.328.7454.1490 15205295 PMC428525

[37] Lewin S, Glenton C, Munthe-Kaas H, Carlsen B, Colvin CJ, Gülmezoglu M, et al. Using qualitative evidence in decision making for health and social interventions: an approach to assess confidence in findings from qualitative evidence syntheses (GRADE-CERQual). PLoS Med. 2015;12(10):e1001895. doi: 10.1371/journal.pmed.1001895 26506244 PMC4624425

