# Supplementary material for: Modifiable determinants of adherence to tyrosine kinase inhibitors in classical myeloproliferative neoplasms: A mixed-methods systematic review protocol
Source: PLoS One. 2026 Sep 18;21(9):e0358485. doi: 10.1371/journal.pone.0358485 (PMC13588520; doi:10.1371/journal.pone.0358485)
Supplement: Supplementary file 1 [file pone.0358485.s001.docx]

**S1 Appendix. Database search strategy**

**S1.1 Appendix -** Search Terms Strategy, Alternatives, and Synonyms for Keywords

**Concept Group 1 (Diseases - related terms)**

(Leukemia OR "Leukemia" OR "Leukemias" OR "Leucocythemia" OR "Leucocythemias" OR "Leucocythaemia" OR "Leucocythaemias" OR " Myeloid Leukemia" OR "Myeloid Leukemias" OR "Leucemia Mieloide" OR "Primary Myelofibrosis" OR "Bone Marrow Fibrosis" OR "Myelofibrosis" OR "Idiopathic Myelofibrosis" OR "Myeloid Metaplasia" OR "Primary Myelofibroses" OR "Bone Marrow Fibroses" OR "Myelofibroses" OR "Myeloid Metaplasias" OR "Nonleukemic Myelosis" OR "Agnogenic Myeloid Metaplasias" OR "Chronic Idiopathic Myelofibrosis" OR "Agnogenic Myeloid Metaplasia" OR "Mielofibrose Primária" OR "Mielofibrosis Primaria" OR Myelosclerosis OR Myeloscleroses OR "Nonleukemic Myeloses" OR "Leukemia, Myelogenous, Chronic, BCR-ABL Positive" OR "Leukemia, Myelomonocytic, Chronic" OR "Chronic Myeloid Leukemia" OR "Chronic Myelomonocytic Leukemia" OR "Chronic Myeloid Leukemias" OR "Chronic Myelocytic Leukemia" OR "Chronic Myelocytic Leukemias" OR "Chronic Myelogenous Leukemia" OR "Chronic Myelogenous Leukemias" OR

"Leukemia, Philadelphia-Positive Myeloid" OR "Leukemias, Philadelphia-Positive Myeloid" OR

"Ph1-Positive Myelogenous Leukemia" OR "Ph1-Positive Myelogenous Leukemias" OR "Chronic Granulocytic Leukemia" OR "Chronic Granulocytic Leukemias" OR "Leucemia Mielogênica Crônica BCR-ABL Positiva" OR "Leucemia Mielógena Crónica BCR-ABL Positiva" OR "Ph1-Positive Myeloid Leukemia" OR "Ph1-Positive Myeloid Leukemias" OR "Philadelphia- Positive Myeloid Leukemia" OR "Philadelphia-Positive Myeloid Leukemias" OR "Leukemia, Myeloid, Accelerated Phase" OR "Leucemia Mieloide de Fase Acelerada" OR "Leukemia, Myeloid, Chronic-Phase" OR "Leucemia Mieloide de Fase Crônica" OR "Leucemia Mieloide de Fase Crónica" OR "Leukemia, Myeloid, Chronic, Atypical, BCR-ABL Negative" OR "Leucemia Mieloide Crônica Atípica BCR-ABL Negativa" OR "Leucemia Mieloide Crónica Atípica BCR- ABL Negativa" OR "Polycythemia Vera" OR "Polycythemias" OR "Osler-Vaquez Disease" OR "Erythremias" OR "Erythremia" OR "Vaquez Disease" OR "Osler Disease" OR Polycythemia OR "Policitemia Vera")

**Concept Group 2 (Medications - related terms)**

("Dasatinib" OR dasatinib OR "BMS354825" OR "Sprycel" OR Dasatinibe OR "Imatinib Mesylate" OR imatinib OR "CGP57148B" OR "ST1571" OR "STI571" OR "Gleevec" OR Glivec OR Imatinibe OR "Mesilato de Imatinibe" OR "Mesilato de Imatinib" OR "Protein-Tyrosine Kinases" OR "Protein Kinase Inhibitors" OR "Inhibidores de Proteínas Quinasas" OR "Inibidores de Proteínas Quinases" OR ITQSS OR "Tyrosine Kinase Inhibitors" OR "Tyrosine Kinase" OR "Inibidores de Proteína Tirosina Quinase" OR "Inibidores de Proteína Tirosina Quinases" OR "Inibidor de Tirosina Quinase" OR "Inibidores de Tirosina Quinase" OR "Tyrosine Protein Kinase Inhibitors" OR "Inhibidores de la Tirosina Proteína Quinasa" OR bosutinib OR "SKI606" OR ruxolitinib OR "INCB-18424 phosphate" OR "Jakafi" OR "Opzelura" OR fedratinib OR "Inrebic" OR "SAR302503" OR "SAR302503A" OR nilotinib OR "Tasigna" OR "AMN107" OR ponatinib OR "AP24534" OR "Iclusig" OR radotinib OR "IY5511HCl" OR

Asciminib OR Scemblix OR "ABL001")

**Concept Group 3 (Adherence - related terms)**

("Treatment Adherence and Compliance" OR "Patient Compliance" OR "Medication

Adherence" OR complian* OR "non-adherence" OR "non-compliance" OR noncomplian* OR nonadheren* OR comply* OR adhere* OR "Cooperação e Adesão ao Tratamento" OR "Cumplimiento y Adherencia al Tratamiento" OR "Cooperación del Paciente" OR "Cooperação do Paciente")

**Source**: Author’s own work (2026)

**S1.2 Appendix -** Unified Search Strategy for CINAHL, Embase®, LILACS, Medline/PubMed,

Web of Science, Cochrane Library, and PsycINFO® databases

|  | **Unified Search Strategy** |  |
| --- | --- | --- |

("Dasatinib" OR dasatinib OR "BMS354825" OR "Sprycel" OR Dasatinibe OR "Imatinib Mesylate" OR imatinib OR "CGP57148B" OR "ST1571" OR "STI571" OR "Gleevec" OR Glivec OR Imatinibe OR "Mesilato de Imatinibe" OR "Mesilato de Imatinib" OR "Protein- Tyrosine Kinases" OR "Protein Kinase Inhibitors" OR "Inhibidores de Proteínas Quinasas" OR "Inibidores de Proteínas Quinases" OR ITQSS OR "Tyrosine Kinase Inhibitors" OR "Tyrosine Kinase" OR "Inibidores de Proteína Tirosina Quinase" OR "Inibidores de Proteína Tirosina Quinases" OR "Inibidor de Tirosina Quinase" OR "Inibidores de Tirosina Quinase" OR "Tyrosine Protein Kinase Inhibitors" OR "Inhibidores de la Tirosina Proteína Quinasa" OR bosutinib OR "SKI606" OR ruxolitinib OR "INCB-18424 phosphate" OR "Jakafi" OR "Opzelura" OR fedratinib OR "Inrebic" OR "SAR302503" OR "SAR302503A" OR nilotinib OR "Tasigna" OR "AMN107" OR ponatinib OR "AP24534" OR "Iclusig" OR radotinib OR "IY5511HCl" OR Asciminib OR Scemblix OR "ABL001") AND (Leukemia OR "Leukemia" OR "Leukemias" OR "Leucocythemia" OR "Leucocythemias" OR "Leucocythaemia" OR "Leucocythaemias" OR " Myeloid Leukemia" OR "Myeloid Leukemias" OR "Leucemia Mieloide" OR "Primary Myelofibrosis" OR "Bone Marrow Fibrosis" OR "Myelofibrosis" OR "Idiopathic Myelofibrosis" OR "Myeloid Metaplasia" OR "Primary Myelofibroses" OR "Bone Marrow Fibroses" OR "Myelofibroses" OR "Myeloid Metaplasias" OR "Nonleukemic Myelosis" OR "Agnogenic Myeloid Metaplasias" OR "Chronic Idiopathic Myelofibrosis" OR "Agnogenic Myeloid Metaplasia" OR "Mielofibrose Primária" OR "Mielofibrosis Primaria" OR Myelosclerosis OR Myeloscleroses OR "Nonleukemic Myeloses" OR "Leukemia, Myelogenous, Chronic, BCR-ABL Positive" OR "Leukemia, Myelomonocytic, Chronic" OR "Chronic Myeloid Leukemia" OR "Chronic Myelomonocytic Leukemia" OR "Chronic Myeloid Leukemias" OR "Chronic Myelocytic Leukemia" OR "Chronic Myelocytic Leukemias" OR "Chronic Myelogenous Leukemia" OR "Chronic Myelogenous Leukemias" OR "Leukemia, Philadelphia-Positive Myeloid" OR "Leukemias, Philadelphia-Positive Myeloid" OR "Ph1- Positive Myelogenous Leukemia" OR "Ph1-Positive Myelogenous Leukemias" OR "Chronic Granulocytic Leukemia" OR "Chronic Granulocytic Leukemias" OR "Leucemia Mielogênica Crônica BCR-ABL Positiva" OR "Leucemia Mielógena Crónica BCR-ABL Positiva" OR "Ph1- Positive Myeloid Leukemia" OR "Ph1-Positive Myeloid Leukemias" OR "Philadelphia-Positive Myeloid Leukemia" OR "Philadelphia-Positive Myeloid Leukemias" OR "Leukemia, Myeloid, Accelerated Phase" OR "Leucemia Mieloide de Fase Acelerada" OR "Leukemia, Myeloid, Chronic-Phase" OR "Leucemia Mieloide de Fase Crônica" OR "Leucemia Mieloide de Fase Crónica" OR "Leukemia, Myeloid, Chronic, Atypical, BCR-ABL Negative" OR "Leucemia Mieloide Crônica Atípica BCR-ABL Negativa" OR "Leucemia Mieloide Crónica Atípica BCR- ABL Negativa" OR "Polycythemia Vera" OR "Polycythemias" OR "Osler-Vaquez Disease" OR "Erythremias" OR "Erythremia" OR "Vaquez Disease" OR "Osler Disease" OR Polycythemia OR "Policitemia Vera") AND ("Treatment Adherence and Compliance" OR "Patient Compliance" OR "Medication Adherence" OR complian* OR "non-adherence" OR "non- compliance" OR noncomplian* OR nonadheren* OR comply* OR adhere* OR "Cooperação e Adesão ao Tratamento" OR "Cumplimiento y Adherencia al Tratamiento" OR "Cooperación del Paciente" OR "Cooperação do Paciente")

Source: Author’s own work (2026)

**S1.3 Appendix - -** Search Strategy for Gray Literature

**Open Gray & Google Scholar**

"Tyrosine Kinase" adherence leukemia myelofibrosis polycythemia

**WorldCat**

("Tyrosine Kinase" OR ITQSS OR dasatinib OR imatinib OR ponatinib OR nilotinib OR

radotinib OR fedratinib OR bosutinib OR ruxotinib) AND (Leukemia OR Myelofibrosis OR Polycythemia) AND (complian* OR comply* OR adhere*)

Source: Author’s own work (2026)
