# Supplementary material for: Modifiable determinants of adherence to tyrosine kinase inhibitors in classical myeloproliferative neoplasms: A mixed-methods systematic review protocol
Source: PLoS One. 2026 Sep 18;21(9):e0358485. doi: 10.1371/journal.pone.0358485 (PMC13588520; doi:10.1371/journal.pone.0358485)
Supplement: Supplementary file 2 [file pone.0358485.s002.docx]

**S2 Appendix. Codebook**

**Introduction to the Codebook**

This codebook was developed by FCI and SG to support the coding process using the Theoretical Domains Framework (TDF) and COM-B model in the context of a systematic review.

Since the coding process depends on the reviewers’ experience and interpretation, it is essential to maintain a consistent and structured approach. To support this, the following rules were established:

1. **Qualitative studies**: **Quotations or themes** extracted from qualitative data may be coded using up to **three TDF domains** when multiple Behavioural constructs are clearly present.
2. **Quantitative studies**: Only **one TDF domain** should be assigned: the one that most directly represents the finding.
3. **Be simple and objective**: Avoid overanalysing the quotation. Focus on the **most prominent domain(s)** rather than listing every possible match.
4. **Always ask yourself**: *Which domain is most relevant to this quotation/theme/statement as a facilitator or barrier to adherence to TKI treatment?*

**Table 1** provides definitions of the 14 TDF domains, examples of barriers and facilitators, and their corresponding components within the COM-B model.
**Table 2** presents practical examples to illustrate how one or more domains may apply to the same quotation.

**S2.1 Appendix - Theoretical Domains Framework: Definitions and examples related to medication adherence in myeloproliferative neoplasms.** Adapted from Cane et al (2012) [20] e Boot et al (2024) [18]

| **TDF domain and definition** | **Determinants (****i.e. facilitators/barriers) of adherence** | **COM-B Category** |
| --- | --- | --- |
| **Knowledge:** awareness of the existence of something | - **Barriers:** lack of knowledge about MPNs, the role of TKIs, or the risks of treatment discontinuation. - **Facilitators:** clear understanding of the disease, the importance of adherence, side effects, and how TKIs work. | Capability (Psychological) |
| **Skills:** ability or proficiency acquired through practice | - **Barriers:** Difficulty performing practical medication-management tasks, such as opening packaging or organising doses. - **Facilitators:** Ability to organise and take medication correctly through skills developed with practice. | Capability (Physical) |
| **Social/professional role and identity:** a coherent set of behaviourals and displayed personal qualities of an individual in a social or work setting | - **Barriers:** patients do not perceive themselves as responsible for treatment; shifts responsibility to the physician. - **Facilitators:** strong sense of personal responsibility and identification with the role of an "active patient." | Motivation (Reflective) |
| **Beliefs about capabilities:** acceptance of the truth, reality or validity about an ability, talent or facility that a person can put to a constructive use | - **Barriers:** feelings of lack of capacity to maintain long-term treatment. - **Facilitators:** confidence in personal discipline and ability to manage side effects and treatment demands. | Motivation (Reflective) |
| **Optimism:** confidence that things will happen for the best or that desired goals will be attained | - **Barriers:** pessimistic belief that the disease will progress regardless of treatment. - **Facilitators:** belief that following treatment correctly will lead to remission and better quality of life. | Motivation (Reflective) |
| **Beliefs about consequences:** acceptance of the truth, reality or validity of outcomes of a behavioural in a given situation | - **Barriers:** fear of side effects, distrust in medication benefits. - **Facilitators:** conviction that adherence prevents relapses and improves survival. | Motivation (Reflective) |
| **Reinforcement:** increasing the probability of a response by arranging a dependent relationship, or contingency, between the response and a given stimulus | - **Barriers:** absence of noticeable clinical benefits, or negative reinforcement when side effects are mentioned. - **Facilitators:** receiving praise from healthcare professionals or family for treatment engagement. | Motivation (Automatic) |
| **Intentions:** conscious decision to perform a behavioural or to resolve to act in a certain way | - **Barriers:** deliberate intention to stop treatment due to fatigue or adverse effects. - **Facilitators:** strong desire to follow the prescribed treatment plan. | Motivation (Reflective) |
| **Goals:** mental representation of outcomes or end states that an individual wants to achieve | - **Barriers:** competing priorities that lead to neglecting medication. - **Facilitators:** goal to achieve deep molecular response or avoid disease progression. | Motivation (Reflective) |
| **Memory, Attention and Decision Processes:** ability to retain information, focus selectively on aspects of the environment and choose between two or more alternatives | - **Barriers:** frequent forgetfulness, unstable routines, uncertainty about whether the dose was taken. - **Facilitators:** use of reminders, pill organisers, or structured decisions about the ideal time to take medication. | Capability (Psychological) |
| **Environmental Context and Resources:** any circumstance of a person’s situation or environment that discourages or encourages the development of skills and abilities, independence, social competence, and adaptive behavioural | - **Barriers:** difficulty accessing medication, fragmented healthcare system, irregular work hours. - **Facilitators:** easy access to hospital or pharmacy, financial support, an environment that supports routine organisation. | Opportunity (Physical) |
| **Social Influences:** interpersonal processes that can cause an individual to change their thoughts, feelings or behaviours | - **Barriers:** negative influence from family or peers who minimise the importance of adherence. - **Facilitators:** support from family, friends, or medical team, consistent encouragement. | Opportunity (Social) |
| **Emotion:** complex reaction pattern, involving experiential, Behavioural and physiological elements, by which the individual attempts to deal with a personally significant matter or event | - **Barriers:** anxiety, depression, or anger related to the disease or medication routine. - **Facilitators:** emotional acceptance of the diagnosis and development of positive emotional coping strategies. | Motivation (Automatic) |
| **Behavioural Regulation:** anything aimed at managing or changing observed or measured actions | - **Barriers:** absence of concrete Behavioural control strategies. - **Facilitators:** use of techniques such as checklists, alarms, or integrating medication into daily routine (e.g., taking it with breakfast). | Capability (Psychological) + Motivation (Reflective) |

TDF: Theoretical domains framework; COM-B: Capability, Opportunity, Motivation-Behavioural. TKI: tyrosine kinase inhibitors. MPN: classical myeloproliferative neoplasms.

**S2.2 Appendix - Practical Examples of TDF Coding (Single or Multiple Domains per Quotation)**

| **Quotation (generalised explanation)** | **Coding (TDF Domains)** | **Facilitator/Barrier** | **Justification for Domain Assignment** |
| --- | --- | --- | --- |
| The patient stores the medication in a safe place and relies on reminders from others or alarms to ensure adherence. | Behavioural regulation; Memory, attention and decision processes | Facilitator | Behavioural regulation is identified through planned storage and the use of prompts/reminders. Memory attention is involved in consistently remembering the medication-taking routines. |
| The patient describes travelling long distances and receiving consistent support from the healthcare team to access medication. | Environmental context and resources; Memory, attention and decision processes; Behavioural regulation | Facilitator | Environmental context reflects accessibility and healthcare support. Memory attention and Behavioural regulation appear through consistent engagement in routines. |
| The patient integrates spiritual beliefs with treatment adherence, demonstrating religious motivation and positive expectation. | Beliefs about capabilities; Optimism; Beliefs about consequences | Facilitator | Religious beliefs may enhance motivation (optimism and capabilities), while consequences are considered through spiritual interpretation of treatment effectiveness. |
| The patient follows the physician’s recommendations and understands the consequences of not adhering to treatment. | Social professional role and identity; Knowledge | Facilitator | Adherence is influenced by trust in the physician’s professional role and by the patient’s understanding of the clinical consequences of non-adherence. |
| The patient adapts medication intake to reduce side effects, demonstrating an understanding of how and when the medication should be taken. | Beliefs about consequences; Knowledge | Facilitator/Barrier | The patient associates medication-taking with timing and food intake to manage side effects, demonstrating awareness of consequences and knowledge of appropriate or inappropriate medication-taking strategies |
| The patient values receiving written information to improve understanding and support adherence behaviour. | Skills; Knowledge | Facilitator | The request for written information reflects a need to improve comprehension and skills and to reinforce knowledge. |
| The patient describes difficulty maintaining a consistent routine due to family responsibilities and exhaustion. | Environmental context and resources; Memory, attention and decision processes; Behavioural regulation | Barrier | Describes challenge in maintaining routine due to environmental burden and emotional fatigue; highlights behavioural regulation difficulties and memory strain. |
| The patient develops a medication-taking routine linked to regular meals. | Behavioural regulation; Memory, attention and decision processes | Facilitator | The routine is linked to consistent mealtimes, reflecting a behavioural strategy and a cognitive association between medication-taking and an established habit. |
| The patient places medication in a visible spot to form a habit of taking it. | Behavioural regulation; Memory, attention and decision processes | Facilitator | Medication placement in a visible spot promotes automatic behavioural (regulation) and memory cues. |
| The patient rationalises skipping a dose occasionally as not problematic, especially in social contexts. | Intentions; Knowledge; Optimism | Barrier | Downplaying missed doses reflects motivational intention, lack of knowledge of risks, and optimism bias. |
| The patient receives reassurance after missing a dose, reducing worry and reinforcing relaxed adherence. | Reinforcement; Knowledge | Barrier | The patient receives reassurance after missing a dose, which reduces concern and reinforces a more relaxed approach to adherence. |
| The patient expresses emotional burden and frustration about the disease, perceiving it as invisible and disruptive. | Emotion; Optimism | Barrier | Feelings of invisibility and frustration align with emotional responses and pessimism about condition impact. |
| Drug characteristics, such as taste or tablet size, affect adherence. | Environmental context and resources | Barrier/  Facilitator | Drug characteristics, such as taste, tablet size, and the specific type of TKI, influence adherence behavioural. These factors represent elements of the environmental context that can either support or hinder treatment adherence, depending on how they are perceived and managed by the patient. |
| The way in which the TKI is prescribed, such as once-daily rather than twice-daily dosing, and the presence of comorbidities may affect adherence. | Environmental context and resources; Behavioural regulation; Memory, attention and decision processes | Facilitator / Barrier | Environmental context and resources include dosing frequency and the complexity of managing the haematological condition and any comorbidities. A simpler regimen, such as once-daily dosing, may facilitate habit formation and adherence through behavioural regulation and memory cues, whereas more complex regimens may hinder consistent medication use. |
| Some patients report irregular medication use or a lack of interest in treatment. | Intentions; Environmental Context and Resources context and resources | Barrier | Irregular medication use or disengagement from treatment may reflect intentional or passive non-adherence. When disengagement is explicitly reported, it may be classified under intentions. |
| Some patients interrupt treatment for a period of time when the reason is unclear or loss of motivation is suspected. | Environmental context and resources | Barrier | Treatment interruption may result from contextual challenges, such as logistical barriers, lack of support, or loss of motivation. When the exact reason is unclear, the finding is conservatively coded under Environmental context and resources in quantitative studies. In qualitative studies, the behaviour may be mapped to both Intentions and Environmental context and resources, depending on the depth of the narrative and the patient’s perspective |

TDF: Theoretical domains framework; COM-B: Capability, Opportunity, Motivation-Behavioural. TKI: tyrosine kinase inhibitors. MPN: classical myeloproliferative neoplasms.
